# Draft Genome Sequence of *Caloramator mitchellensis*, a Thermoanaerobe Isolated from the Waters of the Great Artesian Basin

**DOI:** 10.1128/genomeA.01578-15

**Published:** 2016-02-04

**Authors:** Bharat K. C. Patel, Valentino Setoa Junior Te'o

**Affiliations:** a Microbial Gene Research and Resources Facility, School of Natural Sciences, Griffith University, Brisbane, Queensland, Australia; b School of Earth, Environmental and Biological Sciences, Queensland University of Technology, Brisbane, Queensland, Australia

## Abstract

The genome sequence of *Caloramator mitchellensis* strain VF08, a rod-shaped, heterotrophic, strictly anaerobic bacterium isolated from the free-flowing waters of a Great Artesian Basin (GAB) bore well located in Mitchell, an outback Queensland town in Australia, is reported here. The analysis of the 2.42-Mb genome sequence indicates that the attributes of the genome are consistent with its physiological and phenotypic traits.

## GENOME ANNOUNCEMENT

*Caloramator mitchellensis* strain VF08^T^ (JCM, 1582^T^; KCTC, 57350^T^) was isolated from a bore-water sample collected at the source of a free-flowing, 403-m-deep Great Artesian Bore (GAB) bore well (registered number 22981) situated in Mitchell, a Queensland outback town in Australia (1). The strain was cultured in PL medium under optimal conditions (pH 7.0 and 55°C) and the DNA was prepared by using a modified Marmur’s method (1, 2). The genomic DNA of *C. mitchellensis* strain VF08^T^ was sequenced using MySeq at the Australian Genome Research Facility (AGRF) core facility. A total of 2,443,429 reads totaling 601 Mbp were assembled into 53 contigs using the GS De Novo assembler, version 2.9. The *N*_50_ contig size was 168,891 bp and the largest contig was 336,266 bp. The assembled data of 2.42 Mbp, with an average GC content of 33.6 mol%, were annotated using Prokka, version 1.10 (3). The genome sequence comprises 2,401 putative protein-coding genes, 56 tRNA genes, and 3 rRNA genes (5S rRNA, 16S rRNA, and 23S rRNA). PhyloSift version 1.0.0_02 (4) analysis indicated that 2 of the 10 taxonomically validated members of the genus *Caloramator* (5) (http://www.bacterio.net/caloramator.html) that have been sequenced to date, namely *C. australicus* RC3 (6) and *C. celere* (7), were the most closely related. Further analysis using the online annotation server RAST (8) indicated that carbohydrate utilization genes (243) were present with a slightly lower number of amino acid degradation and synthesis genes (183) also present in the genome. Unlike in *C. australicus*, in the genome of *C. mitchellensis* genes involved in the uptake and transport of ferric ions were absent, and the profile of genes for resistance to toxic ions, such as arsenic, cobalt, zinc, cadmium, copper, and zinc, was also different. In addition, a cluster of 20 genes involved in the utilization of the amino alcohol, ethanolamine, was present in the genome of *C. mitchellensiss* and several members of the family *Clostridiaceae* but absent in the genomes of *C. australicus* and *C. celere*, suggesting that members of the genus *Caloramator* may have a wide range of metabolic differences and potential roles in carbohydrate modification which could be related adaptations to their habitats. The ethanol amino utilization pathway is restricted to a number of human and animal food-poisoning bacteria, but its potential role in *C. mitchelelensis*, a thermophile, will be interesting to elucidate. The draft whole-genome sequence of *C. mitchellensis* strain VF08^T^ will increase our understanding of the physiology and adaptation of microbial life in the Great Artesian Basin of Australia, a deep geothermal subsurface multiaquifer.

### Nucleotide sequence accession numbers.

This whole-genome shotgun project of *C. mitchellensis* strain VF08 has been deposited in DDBJ/EMBL/GenBank under the accession number LKHP00000000. The version described in this paper is version LKHP01000000.
